# A novel *GNAS* mutation in pseudohypoparathyroidism type 1a in a Chinese man presented with recurrent seizure: a case report

**DOI:** 10.1186/s12902-020-00651-z

**Published:** 2021-01-09

**Authors:** Difei Lu, Aimei Dong, Junqing Zhang, Xiaohui Guo

**Affiliations:** Department of Endocrinology, Peking University First Hospital, Beijing, China

**Keywords:** Pseudohypoparathyroidism, *GNAS* gene, Novel mutation, Case report

## Abstract

**Background:**

Pseudohypoparathyroidism is a rare genetic disease characterized by hypocalcaemia and hyperphosphataemia due to the defect to the guanine nucleotide-binding protein alpha subunit (GNAS) gene. Patients with pseudoparathyroidism type 1a and 1c could manifest Albright’s hereditary osteodystrophy and multiple hormone resistance including gonadotropin and thyroid stimulating hormone.

**Case presentation:**

Here we report a Chinese man who presented with fatigue, recurrent seizure and Albright’s hereditary osteodystrophy. His genetic study revealed a heterozygote mutation in the *GNAS* gene [NM_000516.4(*GNAS*): c2787_2788del (p.Val930AspfsTer12)]. After calcium and calcitriol supplement, his seizures achieved partially remission.

**Conclusions:**

We report a case of PHP1a or 1c with a novel frameshift mutation in *GNAS* gene in a patient presenting with AHO, as well as TSH and partial gonadotropin resistance. This mutation in this case has not been reported in literature and adds to the spectrum of genetic mutations related to PHP.

## Background

Pseudohypoparathyroidism (PHP) is a rare disease caused by resistance to parathyroid hormone (PTH), which results in hypocalcaemia, hyperphosphataemia and elevated PTH level. PHP was first reported by Fuller Albright and colleagues in 1942 [1].

PHP is classified into three types based on the defect molecular in the PTH signal transduction pathway, which are named as PHP-1, PHP-2 and pseudopseudohypoparathyroidism (PPHP). Patients with PHP-1 display blunted or no response of urinary cyclic AMP (cAMP) to exogenous PTH administration. Meanwhile, urinary cAMP excretion is normal in PHP-2 patients.

PHP-1 is further divided into three different subtypes 1a, 1b and 1c. Patients with PHP type 1a and 1c exhibit Albright’s hereditary osteodystrophy (AHO) features and other hormone resistance besides PTH. These clinical manifestations are caused by mutations of GNAS gene on chromosome 20q13.2–13.3, which encodes theαsubunit of G protein (Gsα) that combines to various G protein-coupled receptors (GPCR) on cell membranes of target tissues of hormones including PTH/PTHrP, thyroid stimulating hormone (TSH) and gonadotropins. PHP 1b is absence of AHO features and other hormone resistance, and is localized only to the renal tubule that is resistant to PTH.

PPHP differs from other two types in that patients have normal electrolytes homeostasis and the presence of AHO, which typically includes short stature, brachydactyly, obesity, round face and ectopic ossification. In PPHP, patients acquire the mutation from paternal chromosome and have no risk of hormone resistance due to the predominantly maternal expression of Gsα.

We herein report a case of PHP type 1a with a novel mutation in the GNAS gene, in which the patient presented with AHO and multiple hormone resistance.

## Case presentation

A 26-year-old man was born via normal vaginal delivery at full term. He was obese during infancy, and his body weight was as large as 19 kg at the age of 8 months. His body weight was similar to his peers since he reached the age of four. All his milestones were normal except a relatively retarded growth in stature. He was admitted to Peking University First Hospital with recurrent seizures, loss of consciousness, limb and facial rigidity for several seconds to half an hour. He presented mild fatigue between the seizures. There was no significant past medical illness and family history. His father’s height was 1.65 m, and his mother’s height was 1.56 m.

**Fig. 1:**
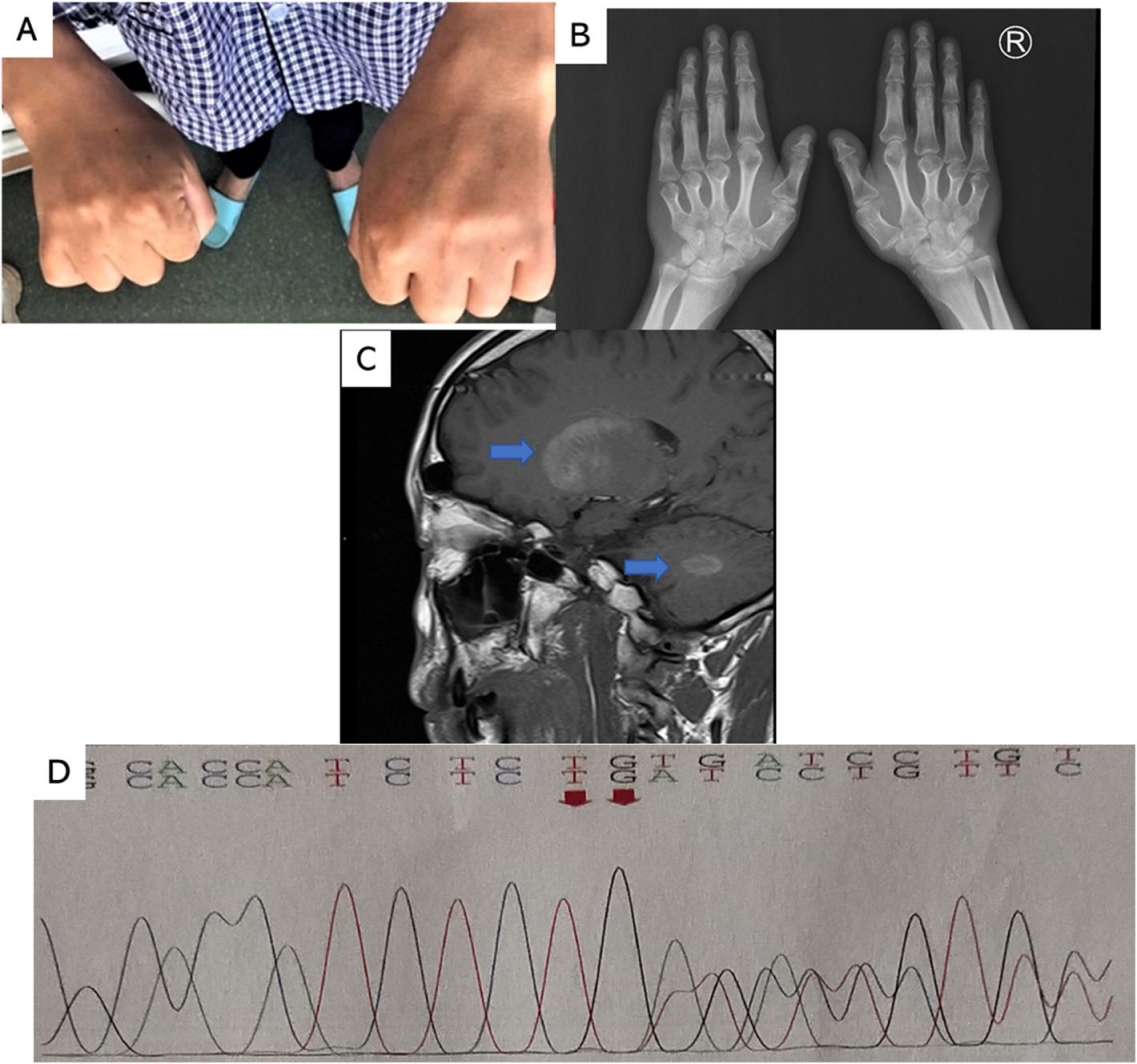
**a** Brachydactyly in bilateral 1st, 3rd, 4th and 5th fingers of the patient as one of the notable signs of AHO. **b** Hands X-ray of the patient showing brachydactyly in bilateral 1st, 3rd, 4th and 5th fingers. **c** Pituitary MRI revealed heterotopic calcification in basal ganglia and cerebellum as pointed by arrows. **d** A novel frameshift mutation in *GNAS* gene of the patient

On physical examination, his body weight was 55.0 kg and height 1.62 m, and his body mass index was 21.0 kg/m^2^. He appeared AHO features of short stature, round face, stocky build and notably brachydactyly in bilateral 1st, 3rd, 4th and 5th fingers (Fig. 1a and b). Tanner stage for his genital development was 4, with a penis length of 4 cm and bilateral testis volume of 20 ml.

During routine examinations, he was noted to have hypocalcaemia with a total serum calcium level of 1.30 mmol/L (normal range, 2.11–2.52 mmol/L) and a serum phosphate level of 1.66 mmol/L (normal range, 0.85–1.51 mmol/L), while the serum PTH level was 705 pg/ml (normal range, 15-65 pg/ml). Pituitary MRI discovered heterotopic calcification in basal ganglia and cerebellum (Fig. 1c), which explained his main complaint of recurrent seizures. Thyroid function showed low T3, T4 and elevated TSH (Table 1). He was negative for anti-thyroid peroxidase antibody (TPOAb) and thyroglobulin antibody (TgAb), with an atrophic thyroid gland in ultrasound scan. Also, he had normal serum testosterone whereas a raised follicle-stimulating hormone (FSH) level (Table 1). Routine test for seminal fluid revealed azoosperia, indicating the diagnosis of primary infertility. Human chorionic gonadotropin (hCG) stimulating test was performed to confirm his testis Leydig cell function, and his serum testosterone elevated from 4.24 ng/ml to 9.45 ng/ml when hCG 2000 IU was intramuscular injected after 72 h, indicating an normal Leydig cell function. Glucose, cortisol and adrenocorticotropic hormone (ACTH) levels were within normal range.

**Table 1:** Laboratory evaluations at the time of diagnosis of the patient

Investigation	Result	Reference range
TSH	21.37μIU/ml	0.55–4.78
Free T4	9.90 pmol/L	11.48–22.70
Free T3	3.77 pmol/l	3.50–6.50
Total T4	55.6 nmol/L	58.1–140.6
Total T3	1.46 nmol/L	0.92–2.79
25-OH-VitD	80.56 nmol/L	75–250
GH (0 am)	2.27 ng/ml	0.03–2.47
LH	9.53mIU/ml	1.24–8.62
FSH	5.20mIU/ml	1.27–19.26
Estradiol	19.0 pg/ml	0–47
hCG stimulating test (hCG 2000 IU intramuscular injection)		
Testosterone (baseline)	4.24 ng/ml	1.75–7.81
Testosterone (24 h)	6.60 ng/ml	
Testosterone (48 h)	6.62 ng/ml	
Testosterone (72 h)	9.45 ng/ml	
Cortisol (8 am)	8.27μg/dl	4.4–19.9
ACTH (8 am)	24.72 pg/ml	7.2–63.3

*TSH* Thyroid-stimulating hormone, *T3* Triiodothyronine, *T4* Thyroxine, *GH* Growth hormone, *LH* Luteinizing hormone, *FSH* Follicle-stimulating hormone, *ACTH* Adrenocorticotropic hormone

The patient was clinically diagnosed as PHP. A mutation analysis on the *GNAS* gene was performed for the patients and his parents and a brother using exon sequencing from peripheral blood leukocytes. His families were found negative, whereas a heterozygous frameshift mutation, NM_080425 (*GNAS*): c.2787_2788del (p.Val930AspfsTer12), in exon 11 of the *GNAS* gene was identified in the patient. This was a novel mutation for PHP-1a, in which the frameshift mutation encoding number 2787_2788 nucleotide led to an evolutionary amino acid change from the valine located 930 to the next 12 amino acids (Fig. 1d).

The patient was diagnosed as PHP 1a or 1c with PTH, TSH and FSH resistance. He was started on calcium and calcitriol replacement as well as thyroxin replacement. His electrolytes, T3, T4 and TSH levels were normal during follow-ups, and PTH reduced to 561.4 pg/ml due to parathyroid glands hyperplasia in accordance with ultrasound scan.

To date, he experienced a mild seizure during 1 year of follow-up, and the symptom of fatigue disappeared. No adverse events happened during treatment. For his infertility, the patients was recommended for genetic counseling for 50% possibility for a child with PPTH, and could be put on intramuscular HMG when serum testosterone was above 3–5 ng/ml for azoosperia. The treatment of calcium and calcitriol supplement partially relieved his recurrent seizures. He no longer felt fatigue with the regular replacement of L-T4 (50μg/d).

## Discussion and conclusions

In this patient, the diagnosis of PHP was delayed due to the subtle symptoms of hypocalcemia and hyperphosphatemia. In this case, the prominent manifestation was seizure resulted from intracranial ectopic calcification, which was a misleading feature at the point of neurologists and could be misdiagnosed as Fahr syndrome [2]. Fahr syndrome is a rare neurodegenerative disease characterized by bilateral basal ganglia calcification, which was common in PHP patients, but the symptoms of the former disease usually occur between the forth and sixth decade of life [2]. Meanwhile, the average age of diagnosis of PHP was between 30 and 50 year-old in some cases [3, 4], since the chronic electrolytes disturbance might not exert evident symptom. Most of the patient was first diagnosed for AHO alone or manifestations associated with ectopic ossification including basal ganglia in this case and subcutaneous calcification. Tetany and epilepsy was reported to be most common symptoms in PHP patients. The prevalence of epilepsy was 47.1, and 94.6% for intracranial calcification with a positive correlation with seizures in a Chinese cohort of PHP [5]. This patient showed no sign of cognitive impairment or mental retardation as is common in PHP-1a patients. The severity of cognitive impairment varies and 30% of patients with PHP-1a display normal cognitive development [6], thus it is critical for clinicians to recognize AHO characteristics including infant obesity and brachydactyly at a small age to achieve early diagnosis.

To date, there is no efficient management for ectopic ossification. Correction of PTH level or serum calcium or phosphate levels does not prevent the occurrence of ectopic ossification [7]. For patients with severe or plate-like heterotopic calcifications in deep tissues or joints of high pressure loads, surgical removal of ectopic ossifications could be recommended. Also, bisphosphonates, which is effective in inhibiting bone turnover, could be put on in such cases to prevent post-surgical recurrences of ectopic ossifications [8].

In our case, PTH, TSH and FSH resistance exhibited at the same time, which induced electrolytes disturbance, hypothyroidism and primary infertility. The patient had a blunted pubertal growth spurt, which was prevalent in PHP-1a patients accompanied with altered gonadal function. Gonadal development was partial impaired in this case, with intact testis Leydig cell function to maintain normal serum testosterone level and azoosperia due to FSH resistance. For boys with PHP-1a, cryptorchidism is common but absent in our case, which could be explained by normal LH function. Female patient with PHP-1a was reported unassisted pregnancy, who had 50% risk of giving birth to offspring with PHP-1a [9]. For further germ cell production, the patient was advised to assisted reproductive treatment of regular HMG intramuscular injection when serum testosterone level was kept under surveillance at the range of 3-5 ng/ml.

As one of the hormones activated via stimulatory G protein –coupled receptors, TSH resistance is highly prevalent in PHP-1a patients. The average level of TSH in PHP-1a patients is 14.1 ± 10.3 mIU/L, ranging from 1.4 mIU/L to 46.0 mIU/L [10]. In this case, the diagnosis of TSH resistance was supported by elevated TSH level, atrophy of thyroid gland and negative screening tests for autoimmune thyroid disease.

GH deficiency due to GHRH resistance could partially explain the final height deficit in patients with PHP-1a, with mean heights of 155 cm in men [11]. In this case, the final height of the patient was 162 cm, and the GH level was in the normal range to provide no evidence for GHRH resistance. Additionally, prolactin deficiency and calcitonin resistance has been previously reported [12, 13], which did not occur in this case. Among the hormones of signal transduction pathway through Gsα, thyroid, gonads, pituitary and renal proximal tubules expresses Gsα only from the maternal allele, and in this way TSH, gonadotropin and GHRH resistance are commonly reported in patients with PHP-1a.

In this case, a novel heterozygous frameshift mutation, NM_080425 (*GNAS*): c.2787_2788del (p.Val930AspfsTer12), in exon 11 of the *GNAS* gene was identified for PHP-1a diagnosis. A heterozygous nonsense mutation in exon 11 of *GNAS* gene (c.857-858delCT) was reported in a 37-year-old female presented with primary adrenal insufficiency and PHP [4]. In a recent review, there have been reported of 176 different germline mutations in the 13 exons of *GNAS* gene that encodes Gsα resulting in PHP-1a [14]. Among them, 44.9% was frameshift mutation as reported by this case. This novel mutation adds to the spectrum of genetic mutations associated to PHP-1, and further completes the genetic diagnosis and contributes to better understanding of these complex disorders.

We report a case of PHP1a or 1c with a novel frameshift mutation in *GNAS* gene in a patient presenting with recurrent seizures due to basal ganglia ectopic calcification and other characteristics of AHO, as well as TSH and partial gonadotropin resistance. This mutation in this case has not been reported in literature and adds to the spectrum of genetic mutations related to PHP.

## Data Availability

The raw date of this study was available from the corresponding author on reasonable request.

## Abbreviations

PHP: Pseudohypoparathyroidism
PTH: Parathyroid hormone
GNAS: Guanine nucleotide-binding protein alpha subunit
AHO: Albright’s hereditary osteodystrophy
PPHP: Pseudopseudohypoparathyroidism
cAMP: Cyclic adenosine monophosphate
Gsα: α subunit of G protein
GPCR: G protein-coupled receptors
TSH: Thyroid stimulating hormone
TPOAb: Anti-thyroid peroxidase antibody
TgAb: Thyroglobulin antibody
FSH: Follicle-stimulating hormone
hCG: Human chorionic gonadotropin
ACTH: Adrenocorticotropic hormone
